# Dietary Upper Gastrointestinal Obstruction Caused by Mushrooms

**DOI:** 10.1002/ccr3.70460

**Published:** 2025-04-18

**Authors:** Yuto Shiozaki, Takuya Otsuki, Kosuke Ishizuka, Yuichi Kato, Kenya Ie, Chiaki Okuse

**Affiliations:** ^1^ Department of General Internal Medicine St. Marianna University School of Medicine Kawasaki Kanagawa Japan; ^2^ Department of General Internal Medicine Kawasaki Municipal Tama Hospital Kawasaki Kanagawa Japan; ^3^ Department of General Medicine Yokohama City University School of Medicine Yokohama Kanagawa Japan

**Keywords:** endoscopy, food impaction, foreign bodies, gastrointestinal obstruction, pyloric obstruction

## Abstract

In patients with a history of peptic ulcer, dietary upper gastrointestinal obstruction at the pylorus may occur. Early endoscopic evaluation should be considered when obstruction is suspected, as timely removal of the foreign body can rapidly improve symptoms and prevent complications.

## Graphical abstract

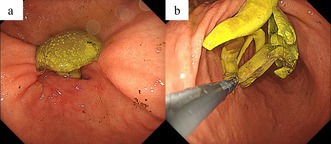

## 1 Case

**FIGURE 1:**
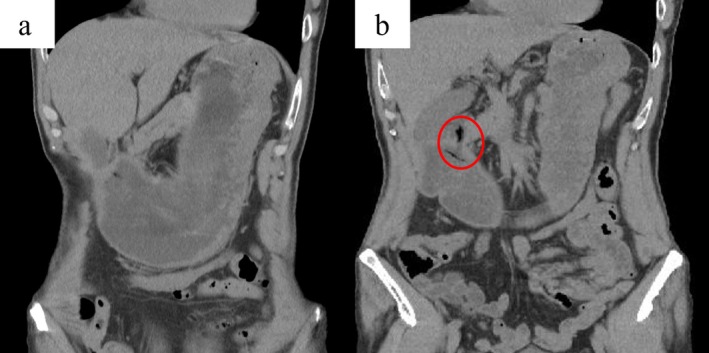
(a) Gastric dilatation, (b) Luminal stricture.

**FIGURE 2:**
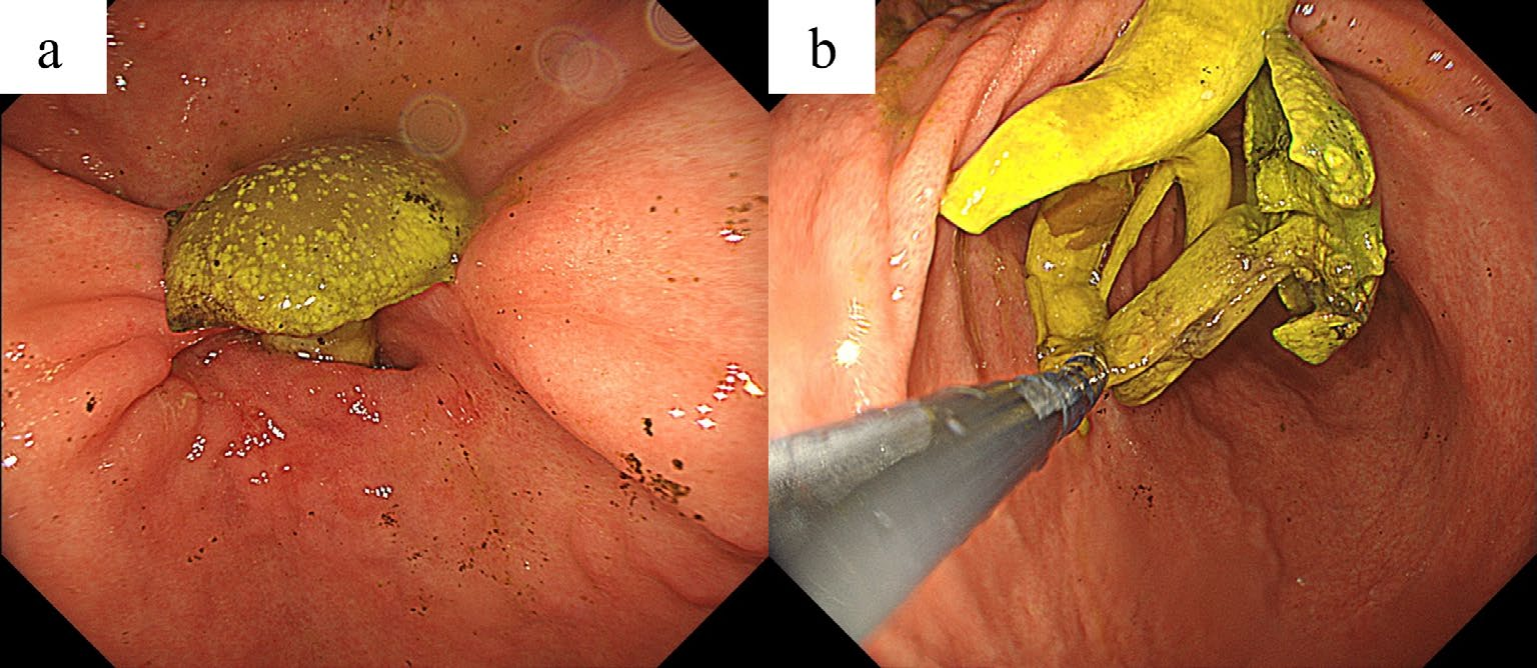
(a) Mushroom shade fitted into the pyloric region, (b) Removal of endoscopes.

A 63‐year‐old Japanese man with a history of duodenal ulcer presented with frequent vomiting after eating and drinking since the previous day. He was taking rabeprazole sodium 20 mg/day. On physical examination, his temperature was 37.3°C, pulse rate was 130 beats per minute, blood pressure was 143/93 mmHg, respiratory rate was 18 breaths per minute, and SpO_2_ was 98% on room air. Laboratory tests revealed an elevated blood urea nitrogen of 30.3 mg/dL and serum creatinine of 2.23 mg/dL, suggesting possible dehydration or acute kidney injury. Abdominal computed tomography revealed gastric dilatation and luminal stricture of the digestive tract extending from the pylorus to the duodenal bulb (Figure 1). The patient was admitted and managed with fasting and intravenous fluid replacement. On the second day of hospitalization, esophagogastroduodenoscopy (EGD) revealed a mushroom‐like foreign body impacted in the pyloric region, along with deformity of the pyloric ring and narrowing of the duodenal bulb, likely caused by a previous duodenal ulcer (Figure 2). Foreign body removal was successfully performed endoscopically. The patient's symptoms, particularly vomiting, improved rapidly, allowing for the gradual reintroduction of food starting from the third day of hospitalization. He was discharged on the 8th day without any recurrence of symptoms.

Upper gastrointestinal obstruction can result from the ingestion of foreign bodies, malignant tumors, or peptic ulcers. The majority of upper gastrointestinal obstructions due to ingestion in adults are diet‐related, most commonly occurring in the esophagus and often at sites of physiologic stricture, with pyloric obstruction being a rare occurrence [1]. Foods that can cause dietary upper gastrointestinal obstruction include meat, fish bones, rice cakes, and gastroliths [2]. For sharp objects, magnets, batteries, or large and long objects lodged in the stomach, EGD within 24 h is recommended to prevent complications [3].

## Author Contributions

**Yuto Shiozaki:** writing – original draft. **Takuya Otsuki:** writing – review and editing. **Kosuke Ishizuka:** writing – review and editing. **Yuichi Kato:** writing – review and editing. **Kenya Ie:** writing – review and editing. **Chiaki Okuse:** writing – review and editing.

## Consent

Written informed consent was obtained from the patient for the publication of this case report and accompanying images.

## Data Availability

The data that support the findings of this study are available from the corresponding author upon reasonable request.
